# Evaluating the Effectiveness of Pretreatment With Intravenous Fluid in Reducing the Risk of Developing Contrast-Induced Nephropathy: A Systematic Review and Meta-Analysis

**DOI:** 10.7759/cureus.24825

**Published:** 2022-05-08

**Authors:** Hany A Zaki, Khalid Bashir, Haris Iftikhar, Mubarak Alhatemi, Amr Elmoheen

**Affiliations:** 1 Emergency Medicine, EgBEM, MRCEM, Hamad Medical Corporation, Doha, QAT; 2 Medicine, Qatar University, Doha, QAT; 3 Emergency Medicine, Hamad Medical Corporation, Doha, QAT; 4 College of Medicine, Qatar University Health, Doha, QAT

**Keywords:** contrast associated nephropathy, systematic review and meta-analysis, observational cross-sectional study, intravenous fluid, contrast-induced nephropathy (cin)

## Abstract

Contrast media administration to patients during cardiac events increases the risk of developing contrast-induced nephropathy (CIN). CIN is among some complications usually associated with the percutaneous coronary intervention and may result in acute renal failure. Several risk factors are associated with CIN. These risk factors include; age (elderly patients), pre-existing renal impairment, diabetes mellitus, and the use of high osmolar contrast media. Studies have shown that several measures such as using low osmolar contrast media, N-acetylcysteine, intravenous sodium bicarbonate, and hydration through oral or intravenous fluid administration play a significant role in CIN incidence reduction. Hydration using intravenous fluid, especially saline solution, has been critical in preventing CIN. Prehydration using the intravenous fluid before contrast media administration is vital.

A systematic literature search with meta-analysis for relevant and original articles was carried out from 2000 to 2022 on databases such as PubMed, Cochrane Library, Google Scholar, ScienceDirect, Web of Science, and Embase. The search on the databases was based on various keywords related to intravenous fluid and CIN. The studies that met the inclusion criteria were critically analyzed, and data such as study design, interventions, participants, and outcomes of the research were retrieved.

Out of the 784 results yielded during the initial search, ten articles met the eligibility criteria and were included in the study. The data analysis obtained from the included studies showed that pretreatment using intravenous fluid has conflicting results. Some studies showed that hydrating patients using intravenous fluid before contrast media administration significantly reduces the risk of CIN. In contrast, others claimed that intravenous fluid has minimal impact on preventing CIN.

Despite the different investigations conducted on CIN, it remains insufficiently understood. From the analysis, most of the studies support that intravenous fluid administration decreases the occurrence of CIN in patients that receive contrast media. The analysis also has established that oral hydration is similar to intravenous fluid administration in reducing CIN incidence.

## Introduction and background

Contrast-induced nephropathy (CIN) is associated with renal function deterioration related to contrast media administration [1]. The understanding of CIN pathogenesis is considered complex and insufficient. However, studies have reported that CIN results from direct toxicity to the renal tubular epithelium, oxidative stress, ischemic injury, and renal tubular obstruction [2-4]. An extensive study has been conducted on the factors that result in increased CIN in patients. First, age is considered a non-modifiable risk factor for CIN. Despite not having sufficient reason, it has been reported that older people are more likely to develop CIN. It is alleged that this increased CIN incidence among the elderly is due to various factors such as; reduction in the glomerular filtrate, tubular function, increased amount of contrast due to increased difficulty of vascular access, presence of multivessel diseases, and comorbid. Previous studies have reported that CIN is highly observed in patients above 70 years [5-7]. Similarly, the other known independent predictor of CIN is diabetes mellitus [7-9]. A previous study reported that CIN incidences are highly observed in diabetic patients with creatinine greater than 4.0mg/dL compared to those with creatinine between 2.0 and 4.0mg/dL [10]. Cardiac factors such as congestive heart failure, anterior MI, cardiogenic shock, and intra-aortic balloon pumps have also been found to increase incidences of CIN after PCI. This increased risk has been associated with all these factors reducing renal perfusion [7,9,11,12]. Additionally, one modifiable risk factor of CIN is the volume of contrast. According to a previous study conducted by McCullough et al., the risk of CIN developing after the procedures is minimized for patients subjected to contrast media of less than 100mL [13].

Pre-existing renal diseases also increase the chances of developing CIN. Recent studies have reported that about 15% of patients with chronic renal impairments scheduled for therapeutic and diagnostic radiographic procedures are more likely to develop CIN [8]. About 0.5% to 12% of the patients with chronic renal impairments usually require dialysis and more extended hospitalization [11]. CIN is generally accompanied by a 0.5mg/dL or more increase in creatinine levels in relation to the baseline levels [8]. Within 24 hours of exposure to contrast media, the rise in creatinine levels is usually detectable, and this increase peaks between three and five days before normalizing after 10-14 days. The contrast media exerts a toxic effect on the tubular epithelial cells, thus resulting in nephropathy and hemodynamic instabilities of the renal blood flow. Iso-osmotic contrast media is associated with less renal tubules injury compared to low-osmolality contrast media. Fact that there is an increase in the number of contrast medium-based procedures among high-risk patients, the incidence of CIN is very relevant in everyday clinal practice. Recent studies have shown that the incidence of CIN in the general public is about 1%-6%, and the rates are even higher in patients undergoing angiography compared to intravenous injection of contrast media [14]. In diabetic nephropathy patients, the incidence rates of CIN are usually as high as 40%-50% [1]. A previous study on the relation between CIN incidence in diabetic nephropathy patients and level of contrast exposure reported that 50% of the patients were observed to have an increase in creatinine levels [15]. Similarly, another study conducted on 1,196 patients reported that 40.8% of renal insufficiency and diabetic patients developed CIN, while only 8.4% of patients without diabetes or renal disease developed CIN.

Moreover, CIN is among the major causes of renal insufficiency developed in the hospital setting [16] and is reported to increase both short-term and long-term mortality [7,9,17,18]. Several pretreatment measures in the past have been used to prevent CIN in patients. These measures include; adequate hydration using intravenous fluids such as isotonic or half-isotonic saline, antioxidant compounds such as N-acetylcysteine (NAC), or ascorbic acid, use of low or iso-osmolar contrast agents, use of sodium bicarbonate, and hemodialysis and hemofiltration. N-acetylacetone has been used to prevent CIN due to its renal vasodilatory and antioxidative properties [19]. The vasodilation mechanism involves the vasodilation of the kidney vessels, thus improving renal hemodynamics [20]. Additionally, NAC exhibits its antioxidant property by scavenging oxygen free radicals hence preventing the direct damage of oxidative tissues in patients undergoing contrast media intervention. Several clinical trials have been conducted to evaluate the benefits of NAC to reduce the risk of developing CIN. For example, a study comparing NAC and saline hydration reported that NAC administered orally was more beneficial in preventing CIN among patients with minor to moderate renal insufficiency scheduled to undergo coronary angiography using low doses of contrast agents [21]. Similarly, another study reported that a doubling NAC dose (1200 mg twice daily for two days) is more effective in reducing the risk of CIN, especially when a non-ionic low osmolality contrast dye is used in large amounts [22].

Pretreatment with sodium bicarbonate is also essential in CIN prevention. Several studies, such as a previous randomized trial on 137 patients with renal insufficiency and scheduled to undergo radiocontrast intervention, indicated that sodium bicarbonate effectively averted CIN development in the patients [23]. The study showed that CIN was less observed in patients pretreated with sodium bicarbonate (one out of 60 patients) than those pretreated with sodium chloride (eight out of 59 patients). The results from the study also suggest that pretreatment with sodium bicarbonate one hour before receiving contrast media injection is essential and efficient in reducing CIN among patients. It is also understood that the type of contrast media to be administered is critical in preventing CIN. A meta-analysis study comparing nephrotoxicity of high and low-osmolar contrast media reported that CIN is more likely to be observed in patients treated using high-osmolar contrast media than those receiving low-osmolar contrast media and with pre-existing renal insufficiency [24]. Similarly, a large randomized trial reported that for patients with renal impairments, the incidence of CIN is 3.3 times more likely to occur in patients receiving high-osmolar contrast media (diatrizoate) than those receiving low-osmolar contrast media (iohexol) [25]. In recent studies, the effect of using low-osmolar contrast media on the risk of developing CIN has been evaluated in patients scheduled to undergo angiography. In a randomized trial conducted on patients with diabetes mellitus and renal function impairment, it is reported that a 3% CIN incidence was observed in patients receiving iso-osmolar contrast media, while 26% of patients in low-osmolar contrast media intervention developed CIN [26]. Additionally, the study reported that no severe case of CIN (serum creatinine [SCr] increase > 1mg/dL or > 88 µmol/L) development was reported in patients that received iso-osmolar contrast media, while 15% severe cases of CIN were reported in patients that received low-osmolar contrast media.

Studies have also been conducted to evaluate the effect of hemodialysis and hemofiltration on CIN development. Studies have claimed that hemodialysis effectively removes contrast media but is not beneficial in reducing CIN’s risk [27,28]. However, in a study conducted on more than 100 patients scheduled to undergo PCI and with chronic renal failure, it was reported that periprocedural hemofiltration effectively prevented renal function from worsening due to CIN [29]. Despite hemofiltration showing a positive effect in reducing the risk of CIN, the study is questionable due to the high mortality rate reported in the control group administered with heparin [30]. Hemofiltration has numerous limitations, including high cost, need for intensive care, and heparin-induced bleeding in the patients [29,31]. Studies have also discussed the effect of pharmacological prophylaxis using calcium channel blockers, dopamine, etc., on reducing the risks of CIN. However, these attempts at pharmacological prophylaxis have turned out to be futile in the reduction of the CIN incidence, and currently, they are not recommended measures for preventing CIN.

Out of all these measures that decrease the chances of developing CIN, hydration regimens are more considered to avert incidences of CIN. Dehydrated patients are associated with decreased renal blood flow and glomerular filtration rates [30]. Therefore, hydration before contrast media is essential in augmenting renal blood flow and glomerular filtration. Studies have reported that pretreatment with intravenous fluids, especially isotonic solution, reduces the risk of CIN [32,33]. The primary aim of this systematic review and meta-analysis is to demonstrate whether pretreatment with intravenous fluid reduces the risk of developing CIN.

## Review

Literature search and reporting

This systematic review and meta-analysis were conducted per Preferred Reporting Items for Systematic Reviews and Meta-analyses (PRISMA) guidelines. Six online databases were scoured for original and relevant studies under priori protocol from PROSPERO. These online databases include; PubMed, Cochrane Library, Google Scholar, ScienceDirect, Web of Science, and Embase.

Searches

For an enhanced and effective search, specific keywords and Boolean operators “AND” and “OR” were used. The search criteria used were as follows; (IV OR intravenous fluid OR isotonic fluid OR half isotonic fluid OR saline) AND (reduce OR Prevent) AND (CIN OR Contrast-induced nephropathy) AND (RCT OR cross-sectional study OR cohort study). The reference lists from the identified relevant literature were scoured for additional studies. The studies retrieved and included for the analysis in this systematic review and meta-analysis were published between 2000 and 2022. This publication range period ensured that the information retrieved from these studies was relevant and up to date.

Eligibility criteria

This systematic review and meta-analysis applied the inclusion and exclusion criteria to identify relevant studies to be included. The inclusion criteria used for study inclusion were as follows: studies that were published in the English language, studies that evaluated the effect of any Intravenous fluid in reducing the risk or preventing CIN, the studies that were only conducted on human beings were included from 2000 to 2022, and the studies that evaluated their data on more than 10 patients were included.

Studies were excluded from this research if they were published in languages other than English. Translated documents were also excluded since some scientific terms are difficult to translate or may have a different meaning. Descriptive studies and other systematic reviews were not eligible for inclusion. Additionally, studies that evaluated other pretreatment measures other than intravenous fluid in CIN prevention were excluded.

Data extraction, quality assessment, and synthesis

Two reviewers were tasked with screening articles based on the inclusion and exclusion criteria. Studies that met inclusion criteria were critically analyzed, and relevant data was retrieved using the PICO guidelines. The data retrieved included author, year of publishment, study design, sample size, the intravenous fluid used, and outcomes. The inconsistencies that arose during the data extraction process were resolved through consultation with the third reviewer. Cochrane Revman software was used for statistical and descriptive analysis of the articles. Additionally, the quality of included articles was analyzed using the research and quality scoring method; the sum of the combined score of all rating metrics was used in the ultimate rating of the included studies. Every article included in this study was rated from 0 to 9, subject to the perceived quality of the article. Low-quality studies had a final score of 0-5, while high-quality studies had a score of 6-9. Despite the scores showing the quality of the study, they failed to guarantee faithfulness. Similarly, the quality and level of confidence of included studies were done by applying the Grading of Recommendations Assessment, Development, and Evaluation (GRADE). The Cochrane risk of bias tool was also used to examine the reliability and transparency of the retrieved data and information from the included studies.

Study selection

**Figure 1:**
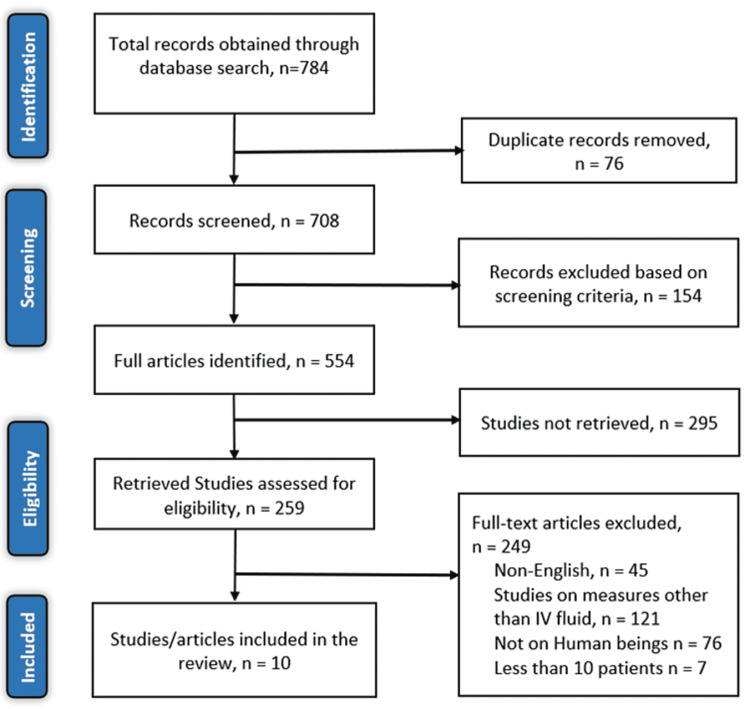
PRISMA flow diagram of the literature search results PRISMA: Preferred Reporting Items for Systematic Reviews and Meta-Analysis

In our initial search through the online databases using the mentioned keywords, we identified a total of 784 relevant articles. After screening for duplicate articles by the two reviewers, 76 articles were eliminated. Out of the remaining 259 articles that could be retrieved fully, 249 articles were excluded since they did not meet the eligibility criteria. Forty-five articles were published in languages other than English, 121 articles evaluated other pretreatment measures that reduce the risk of developing CIN, 76 studies evaluated the effect of intravenous fluids in reducing the risk of developing CIN in animals and seven studies had less than 10 patients (Figure 1), PRISMA flow chart illustrating the search strategy and included studies.

Study characteristics

The characteristics of included studies specifying study design, outcomes, and interventions are given in Table 1.

**Table 1:** Characteristics of included studies specifying study design, outcomes, and interventions CIN: contrast-induced nephropathy; NAC: N- acetylcysteine; PCI: percutaneous coronary intervention; NaCl: sodium chloride; SCr: serum creatinine; eGFR: estimated glomerular filtrate; CT; computerized tomography

Author ID	Study design	Participants	Intervention	Outcomes
Mueller et al. [33]	Randomized trial	The study was conducted on 425 patients that were scheduled for percutaneous coronary intervention (PCI)	Intravenous and Oral fluids (isotonic saline or half-isotonic saline)	Contrast-Induced Nephropathy (CIN) only developed in 6 out of 415 patients (1.4%). Subgroups such as women, elderly, and diabetic patients had a low risk of developing CIN, i.e., 3.7% of women, 2.3% of elderly patients, 2.9% of diabetics, and patients with stage III kidney disease and Glomerular Filtration Rate (GFR) below 60 ml/min/1.73m2 (4.7%).
Wrobel et al. [14]	Randomized controlled study	102 patients (44 women and 58 men) with cardiovascular disease and diabetes undergoing coronary angiography and/or angioplasty participated.	Intravenous isotonic 0.9% saline fluid and oral hydration	CIN development was observed in 5 patients, i.e., 3 (4.77%) in the intravenous saline group and 2 (4%) oral hydration group. There was no influence on ion parameters in either saline or oral hydration group. There was a significant change from baseline values for serum sodium and potassium levels.
Traub et al. [34]	Randomized placebo-controlled study	The study was conducted on 399 patients scheduled for chest, abdominal, or pelvic Computed Tomography (CT) scans. All the patients were above 18 years.	N-Acetylcysteine plus intravenous fluid (standard saline solution) and Intravenous fluid (saline solution)	7.3% of the patients (26 of 357) developed CIN. CIN incidence in the N- Acetylcysteine (NAC) plus intravenous fluid group was similar to the CIN incidence in the Intravenous fluid group, i.e. (14/185 [7.6%] versus 12/172 [7.0%]. No renal therapy was required for any patients after the follow-up period. There is no significant association between age, N-acetylcysteine, congestive heart failure, and development of CIN. Intravenous fluid administration reduced the risk of developing CIN by 69%.
Castini et al. [35]	Randomized study	The study was conducted on 156 patients above 18 years and with stable serum creatinine levels of ≥1.2 mg/dL.	Intravenous fluid (saline solution), N-Acetylcysteine plus intravenous fluid (saline solution), and intravenous sodium bicarbonate	The total number of patients that developed CIN was 23. Out of the 23, the CIN incidence in the intravenous fluid group was 14% (7 patients), 17% in the intravenous saline plus NAC group (9 patients), and 14% in the intravenous sodium bicarbonate group (7 patients). After a follow-up period of 24hours, a significant decrease in serum creatinine levels from baseline values was observed in both groups.
Mueller et al. [36]	A prospective, randomized, controlled, open-label study	The study was conducted on 1620 patients required to have an elective or emergency coronary angioplasty	Intravenous isotonic solution (0.9% saline) and intravenous half-isotonic solution (0.45% Sodium Chloride (NaCl) plus 5% glucose)	CIN development was observed in 5 participants from the intravenous isotonic group and 14 patients from the intravenous half-isotonic group. For patients in elective procedures, isotonic infusion before the procedure significantly reduces the CIN. Small changes were observed in serum creatinine levels in all groups.
Kong et al. [37]	Randomized clinical trial	The study was conducted on 120 patients undergoing coronary angiography or angioplasty.	Oral fluid hydration (water) and intravenous fluid hydration (normal saline)	CIN was developed by 7 patients (5.8%) after coronary procedures. Out of the 7 patients who developed CIN, 2 (5%) were in group A (intravenous fluid hydration), 3 (7.5%) in group B (Oral hydration group 1 consuming 500ml of water), and 2 (5%) in group C (oral hydration group 2 consuming 2000ml of water). A small increase in serum creatinine level was observed in each group, but the increase did not reach statistically significant levels.
Soliman et al. [38]	Cross-sectional observational study	200 patients scheduled for diagnostic coronary angiography participated	Oral fluid hydration (500ml water) and intravenous fluid hydration (0.9% isotonic saline solution)	A total of 13 patients developed CIN, i.e., 6 patients in the orally hydrated group and 7patients in the intravenous fluid hydration group. None of the patients in either group showed a significant change in creatinine levels. There was no statistically significant change in estimated Glomerular Filtration Rate (eGFR) after the contrast intervention.
Shilbayeh [39]	Cohort study	The study was conducted on 60 patients in a single Saudi Center scheduled for coronary angiography.	Intravenous sodium bicarbonate and intravenous fluid (Normal Saline)	The incidence of CIN development at 24hours and 48hours was 16.7% and 15%. Low CIN development incidences were observed in the saline group compared to the sodium bicarbonate group, i.e., 30% in the saline group versus 38% in the sodium bicarbonate group. There was a significant reduction in potassium level (after 24 hours), eGFR (after 24 and 48 hours), and Creatinine clearance (after 24 hours) in group A (intravenous saline hydration group)
Maioli et al. [40]	Prospective randomized, open-label study	The study was conducted on 1226 patients scheduled for angiographic procedures.	Intravenous sodium bicarbonate and intravenous fluid (0.9% isotonic saline solution)	CIN was developed by a total of 54 patients, i.e., 29 (11.5%) were in the intravenous saline group while 25 (10%) were in the sodium bicarbonate group. No statistically significant difference was observed in the creatinine concentration from the baseline values after 10 days in both groups.
Koc et al. [41]	Prospective controlled trial	The study was conducted on 220 patients with mild to moderate renal dysfunction scheduled for coronary angiography or percutaneous coronary intervention (PCI). The participants were above 18 years and had a creatinine clearance of ≤60 mL/min and/or baseline serum creatinine level (SCr) ≥1.1 mg/dL.	Intravenous N-acetylcysteine plus high dose hydration, high-dose intravenous fluid, and standard intravenous fluid hydration.	CIN occurrence was higher in the high-dose hydration group than the other groups, i.e., 12.5% high-dose hydration group, 5% control group, and 2.5% NAC plus high-dose hydration group.

Meta-analysis

Risk of Bias Assessment

**Figure 2:**
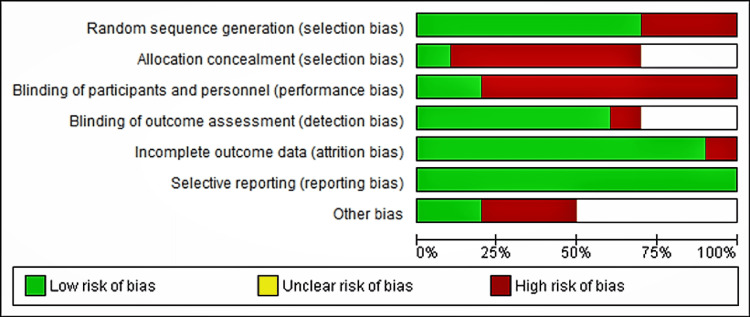
Risk of Bias Graph

Selection, performance, detection, attrition, and reporting bias are the items from the included studies that were evaluated for the risk of bias (Figure 2).

**Figure 3:**
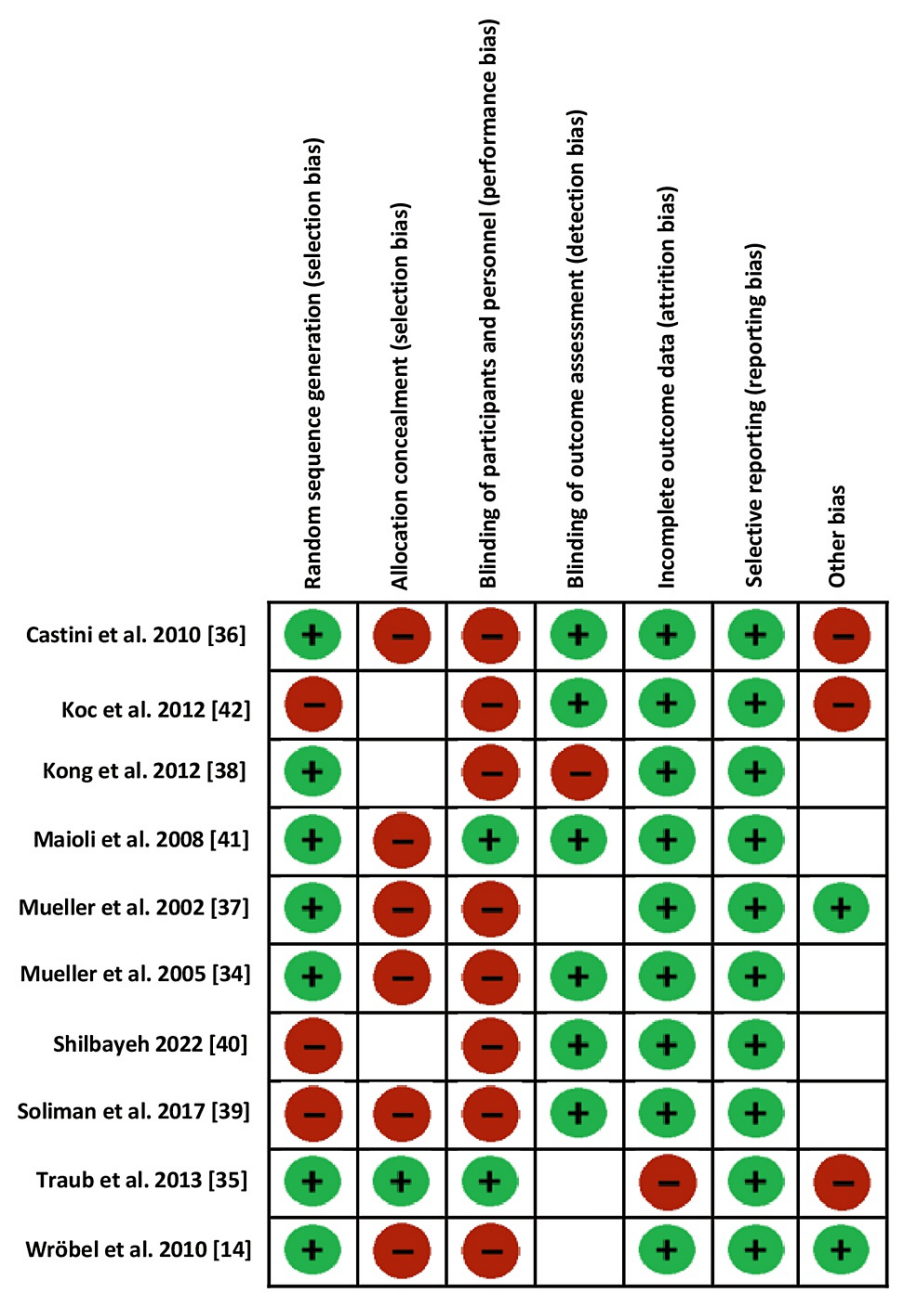
Risk of bias summary

The risk of bias summary figure provides the reviewers’ judgment on the independent risks for each study included in this systematic review and meta-analysis. Low risk is represented by a green circle, while high risk is represented by a red circle. The unclear risk means the lack of clear judgment due to the few details provided in the article (Figure 3), which shows the risk of Bias Summary. 

Plots

**Figure 4:**
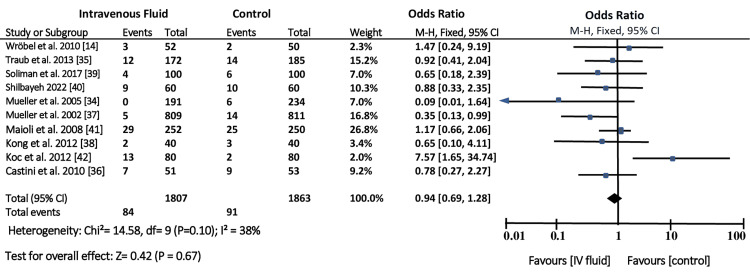
Forest plot for the incidence of contrast-induced nephropathy compared to the other control measures for reducing the risk of contrast-induced nephropathy development

A meta-analysis was conducted on all the studies to evaluate the incidence of CIN in patients who were pretreated with intravenous fluid compared to the incidence of CIN in patients who were pretreated using other measures. Figure 4 shows a forest plot for the incidence of CIN compared to the other control measures for reducing the risk of CIN development.

**Figure 5:**
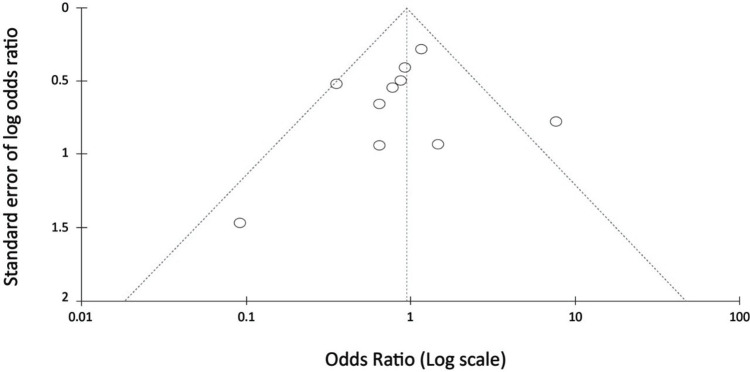
Funnel plot for the incidence of contrast-induced nephropathy compared to the other control measures for reducing the risk of contrast-induced nephropathy development

Forest plot of the 10 included studies illustrating the effectiveness of intravenous fluid in reducing the risk of CIN development. The Review Manager software analysis shows that the studies have little heterogeneity with an inverse variance (I2) of 38%, p = .10. Overall, pretreatment with intravenous fluid reduced the risk of CIN development compared to the other pretreatment measures. The funnel plot is in Figure 5, which shows the incidence of CIN compared to the other control measures for reducing the risk of CIN development.

Many studies support the finding that intravenous fluid reduced the risk of CIN development compared to the other pretreatment measures. 

Discussion

Various studies have evaluated the importance of intravenous fluids in reducing CIN incidence. The forest plot shows that the included studies have conflicting results. Some studies have shown that treating patients with intravenous fluids reduces the risk of developing CIN. In contrast, other studies have reported that intravenous fluids have mild or no significant impact on lowering CIN.

Evidence Shows Intravenous Fluid Significantly Reduces the Risk of Developing CIN

Several studies included in this study have shown that hydration using intravenous fluid is critical in reducing the risk of developing CIN in patients after undergoing contrast media intervention. For example, a study by Kong et al. [37], which evaluated the effectiveness of hydration using both oral and intravenous fluid administration, reported that patients who received intravenous fluid (normal saline at 1mL/kg/h) 12 hours before the coronary angiography or percutaneous coronary intervention (PCI) procedure, blood urea nitrogen (BUN), and SCr significantly decreased after the procedure, i.e., at baseline BUN in patients receiving intravenous fluid administration was 7 3.24mmol/L but at 12 hours, two days and three days after the patients underwent the procedures BUN levels reduced to 7 ± 2.32mmol/L, rose to 8 ± 2.09mmol/L and decreased to 6 ± 2.89mmol/L respectively [38]. On the other hand, SCr levels at the initial follow-up period of 12 hours rose from a baseline of 102 ± 25.90µmol/L to 115 ± 26.89 mmol/L. However, the study claims that this rise did not reach a significant level for developing CIN. After a two- and three-day follow-up period, the SCr levels in the intravenous fluid group decreased to 112 ± 27.32µmol/L and 108 ± 25.22µmol/L. Similarly, a study that evaluated the efficacy of sodium bicarbonate and intravenous saline in reducing the risk of CIN development seemed to support that intravenous saline effectively reduces BUN and SCr levels. The patients in this study underwent pretreatment with intravenous fluid (normal saline) at a rate of 60-70mL/h for six hours before contrast media injection [40]. At baseline, the patients in the saline group had SCr and BUN levels of 93.3µmol/L and 6.8mmol/L, respectively. After contrast media injection and within 24 hours follow-up period, SCr was reduced to 91.9 µmol/L, and BUN level decreased to 6.5mmol/L. However, during a 48-hour follow-up period, the was no statistical difference in the SCr and BUN levels compared to the baseline values. Similarly, another study that compared the benefits of NAC plus intravenous fluid and intravenous fluid alone in reducing the risk of CIN development in patients undergoing emergency computed tomography reported a significant decrease in creatinine levels in patients in the intravenous fluid group [35]. According to the study, the absolute change in creatinine level in the patients who received 500mL of saline solution 30 minutes before contrast administration was -0.025 (0.23) (mean difference in groups of 0.025; 95% CI -0.025 to 0.075). Castini et al. [35] also reported that after the contrast exposure in 24 hours follow-up period, the patients that intravenously received 0.9% isotonic solution at a rate of 1mL/kg, the creatinine levels decreased significantly, i.e., from 1.49 ± 0.30 to 1.37 ± 0.33mg/dL, p = .001 [36].

Additionally, a previous study that evaluated the benefits of intravenous fluid administration (5% dextrose and 0.5% normal saline administered 12 hours before catheterization) reported that there is a significant decrease in BUN and SCr level due to the intravenous fluid administration and reported that no kidney function worsening was observed [42]. Another study that evaluated the relationship between oral and intravenous fluid administration and renal function in diabetic patients undergoing PCI reported that intravenous fluid administration significantly improves renal function. The patients were initially subjected to 1mL/kg/h of 0.9% isotonic solution six hours before the PCI procedure [14]. At baseline, the renal function parameters such as creatinine clearance and urea were 70.33 ± 21.215mL/min and 49.01 ± 23.54mg/dL. After a 72-hour follow-up period, there was a significant improvement in the renal function parameters, i.e., creatinine clearance decreased to 65 ± 23.389mL/min, and Urea increased to 55.62 ± 30.886mg/dL. Improving these renal functions is important as they mean that renal blood flow and glomerular filtration are improved, reversing the hemodynamic conditions resulting in CIN development. A recent study that evaluated the efficacy of intravenous fluid (isotonic solution at a rate of 1 mL/kg/h) reported that the incidence of CIN was found in 11% of the patients in the isotonic saline group as opposed to 21% of patients not receiving any form of hydration [43]. Similarly, a study included in this review and conducted on patients undergoing PCI seemed to have similar results. The patients were subjected to 0.9% isotonic saline and 0.45% saline plus 5% glucose at an infusion rate of 1mL/kg before the procedure. The study reported that out of 425 patients, none of the patients in the isotonic hydration group (n = 191) developed CIN, while in the half-isotonic group, only six patients (2.6%) developed CIN [34]. Another study that compared intravenous sodium bicarbonate and normal saline solution reported that the incidence of CIN in patients from the saline group was much lower compared to those from the sodium bicarbonate group, i.e., 24 hours after PCI procedure, CIN incidence in sodium bicarbonate group versus saline group was 30% to 3.6% while after 48-hour follow-up the difference was 38% to 3.3% [40]. In addition, another study reported that seven patients developed CIN 48 hours after coronary procedures. Out of the seven patients, intravenous fluid administration accounted for the least patients with CIN, i.e., two patients (5%) [38].

Evidence That Intravenous Fluid Has Mild/No Significant Effect on the Reduction of CIN Development

Studies have also shown that the impact of pretreatment with intravenous fluid on reducing the risk of CIN development is low compared to other pretreatment measures. For example, according to a study conducted by Koc et al. [41], it is reported that for patients in the high-dose intravenous fluid administration group, the incidence of CIN was higher as opposed to the NAC plus high-dose hydration group, i.e., 13 out of 80 patients in high dose hydration group developed CIN while only two out of 80 patients in NAC plus high-dose hydration group developed CIN [44]. Similarly, a study conducted by Traub et al. reported that for patients that had SCr levels greater than 1.2mg/dL, CIN was observed in none of the patients that received NAC plus saline, while 7.5% of patients pretreated with saline solution alone developed CIN [35].

In contrast to the studies reporting that intravenous fluid reduces the risk of developing CIN, other studies have shown that pretreatment with intravenous fluid has little or no significant impact on CIN prevention. According to one previous PRECORD study conducted on 201 patients and in which an ionic low osmolar radiographic contrast agent was used during PCI, the procedure reported that a slight improvement in creatinine level was observed in patients that were in the saline group (1.07mL/min) compared to the patients in the control group (0.91mL/min) [45]. Similarly, a study included also claimed that changes that occurred in SCr levels were negligible in all patients. For example, according to the study at 48 hours follow-up period, SCr levels among women increased by a small value of 0.04mg/dL in the isotonic group while an increase of 0.10mg/dL was observed in the half-isotonic groups. Similarly, for patients subjected to a 250mL or more contrast, it was observed that SCr increased by 0.05mg/dL in the isotonic group, while in the half-isotonic group, the increment was 0.08mg/dL. Additionally, a previous randomized study reported that overnight pretreatment with intravenous fluid in patients with moderate renal insufficiency and undergoing contrast media intervention showed no change in SCr levels after 24- and 48-hour follow-up periods. The study reported that out of 37 who received bolus hydration, four patients developed CIN [46]. This incidence suggests a need for patients undergoing angiography and at risk of developing to have a precise hydration therapy.

Limitations of the study

Similar to all the other systematic reviews and meta-analyses, this study was bound to encounter limitations. The primary limitation of our study is that it only included studies that were published in English. This could lead to the omission of some relevant information on the same topic but written in different languages. This review also included some observational design studies, hence making it difficult to identify the impact of intravenous fluid administration in reducing CIN development among the patients observed in these studies. In this systematic review, we have compared studies with different baseline characteristics, which could introduce bias to our study. This review also included a study that conducted its research on low-risk patients; hence it is possible that the CIN incidence reported in this study could have been biased hence introducing bias to our study when comparing this incidence with other studies.

## Conclusions

Despite the different investigations conducted on CIN, it remains insufficiently understood. From the analysis, most of the studies support that intravenous fluid administration decreases the occurrence of CIN in patients that receive contrast media. However, to understand the effectiveness of intravenous fluid, physicians should understand the risk factors associated with CIN development before administering the intravenous fluid. This is very important in ensuring that the appropriate volume of intravenous fluid is administered to patients at a high risk of developing CIN. In most cases, SCr level has been used as the indicator for CIN development; however, SCr level factors in muscle production of creatinine and renal excretion. Therefore, estimated glomerular filtrate (eGFR) should be considered an indicator for CIN development since it can assess the baseline renal function more accurately.

Additionally, eGFR is essential in helping physicians decide what measures should be undertaken to reduce the risk of CIN development. In most cases, intravenous fluid hydration is recommended for patients whose eGFR is less than 30mL/min. This review has also established that oral hydration has similar results to intravenous fluid administration in reducing CIN. Therefore, physicians can encourage the patients to drink fluids and salts before contrast media administration for volume expansion.
